# Patients' choice preferences for specialist outpatient online consultations: A discrete choice experiment

**DOI:** 10.3389/fpubh.2022.1075146

**Published:** 2023-01-05

**Authors:** Mengqiu Wu, Yuhan Li, Chengyu Ma

**Affiliations:** ^1^Key Laboratory of Carcinogenesis and Translational Research (Ministry of Education/Beijing), Laboratory of Genetics, Peking University Cancer Hospital and Institute, Beijing, China; ^2^School of Public Health, Capital Medical University, Beijing, China

**Keywords:** internet hospitals, specialist outpatient online consultations (SOOC), preference, discrete choice experiment, logit regression

## Abstract

**Background:**

Internet hospitals are multiplying with solid support from the Chinese government. In internet hospitals, specialist outpatient online consultations (SOOC) are the primary services. However, the acceptance and utilization rates of this service are still low. Thus, the study of patients' choice preferences for SOOC is needed.

**Objective:**

To analyze the choice preference of patients' SOOC *via* a discrete choice experiment, understand the influence of each factor and promote the development of internet hospitals.

**Methods:**

*Via* a discrete selection experiment, a total of 162 patients from two general hospitals and three specialized hospitals in Beijing were selected for the questionnaire survey. The choice preferences were analyzed by conditional logit regression.

**Results:**

From high to low, patients' willingness to pay (WTP) for the attributes of SOOC is as follows: doctors' recommendation rate (*β*_*highly recommend*_ = 0.999), the convenience of applying SOOC services (*β*_*Convenient*_ = 0.760), the increasing ratio of medical insurance payment for online services compared to offline (*β*_*Increase by* 10%_ = 0.545), and the disease's severity (*β*_*severe*_ = −3.024). The results of the subgroup analysis showed differences in patient choice preference by age, whether the patients had chronic diseases, income, and medical insurance types.

**Conclusion:**

Both price and nonprice attributes influence the choice preference of SOOC for patients. Among them, patients are more inclined to choose SOOC when doctors highly recommend it, when it is convenient to apply, when medical insurance increases by 10%, and when disease severity is mild. The current findings show the government and medical institutions formulate auxiliary policies and welfare strategies by clarifying core attributes and adjusting the levels of different attributes to improve patients' acceptance of SOOC. The utility of SOOC and the further development of internet hospitals are radically promoted.

## Introduction

With the background of coronavirus disease-2019 (COVID-19), more and more people use online consultations of internet hospitals. Internet hospitals are a new medical healthcare service in China and are also the public facilities for doctors to conduct diagnosis and treatment activities based on the internet. Since 2015, the Chinese government has begun to positively publish the policy to motivate the development of internet hospitals as part of “Healthy China”. This policy stimulated the enthusiasm of brick-and-mortar hospitals to establish internet hospitals, leading to a rapid increase in internet hospitals (1). Generally, internet hospitals provide subsequent treatment services such as online consultations, prescriptions, and family doctor services for patients. Among them, specialist outpatient online consultations (SOOC) are the primary service. Usually, patients diagnosed with common illnesses and chronic diseases in offline hospitals can utilize the internet hospitals' resources to receive subsequent treatment from doctors online.

Internet hospitals bring significant benefits, including improved healthcare quality, efficiency and cost containment, especially for chronic patients (2). China is the country with the second-highest investment in telemedicine systems, but internet hospitals' acceptance and utilization rates are still low (3). A better understanding of the shortcomings of SOOCs is important. This study chooses Beijing as the sample since the most reputable Chinese internet hospitals are concentrated mostly in this city. However, in research on 2021 Beijing Medical Insurance Bureau statistics, only 300,000 visits from 32 Beijing internet hospitals are found, which means there are only 20 appointments per day in each hospital (4). Accordingly, the utilization rate of Beijing internet hospitals is low. Previous studies found that patients' choice preferences may affect internet hospitals' utilization rate and acceptance, and the disease severity, as well as socio-demographic factors such as age and income, can have an impact on patients' choice preferences (5–7). Another study also found that the availability and flexibility of online consultation platforms also affect patients' choice preferences (8). Thus, understanding the variable of patients' choice preferences is vital to expanding the number of internet hospitals.

Researchers have analyzed different views in a previous study of patients' choice preferences in online consultations. In summary, these views are primarily concentrated on two aspects: the influence of patients' attributes on health-seeking behavior and the influence of external conditions on health-seeking behavior. Patient attributes mainly involve age, gender, income, disease severity, and medical awareness (9–11). External conditions mainly entail social networks, family factors, treatment distance, price, and medical insurance. The external conditions faced by patients can be further divided into those of medical institutions and social factors (12). At present, most existing research focuses on market surveys of SOOC preferences (13–15). At the same time, there are few studies analyzing patients' preferences and willingness to pay (WTP) against the unique background of internet hospitals in China. Measuring the effect of each attribute and the advantage of using internet hospitals are still the primary concerns. Therefore, this study will research the patients' choice preference for SOOCs and the influence on each attribute.

Based on China's unique internet hospitals model, this study will use the discrete choice experiment (DCE) method to analyze the patient group's choice preference and WTP and put forward targeted strategies and policy suggestions to promote the use of SOOCs by patients and promote the further the development of SOOCs.

## Method

### DCE methodology

**Figure 1:**
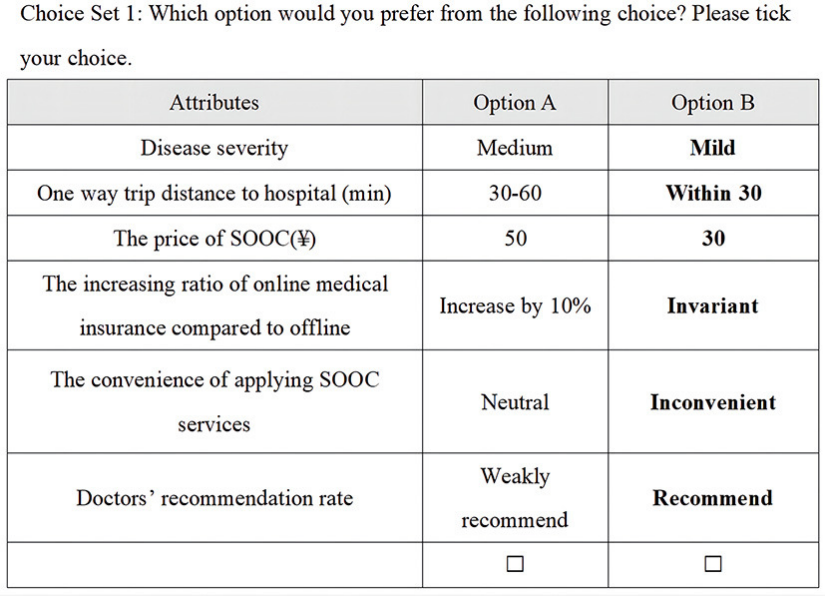
Example choice set: DCE Question 1.

This study used a survey that included a DCE, an econometric method based on random utility theory that simulates a reasonable, direct, and realistic decision-making process by studying the influence of different attributes on respondents' choices (16, 17). In this study, DCE first set up various groups of attributes of SOOCs. Then, DCE elicited patient preferences for various attributes and quantified the value of these attributes by estimating the WTP. For instance, patients need to consider their choices comprehensively and balance these attributes. Each scenario contains different levels of predefined attributes (see, for example, Figure 1). Then, the discrete model estimates the parameters of different attributes through many aspects, such as measuring the value of each attribute by setting continuous variables, including price in a questionnaire, measuring the importance of each relative factor through regression coefficients, and analyzing the different preferences from different groups by stratification or grouping.

### Attribute and level design

According to the specific problems studied, DCE confirms the main attributes and levels that affect the choice behavior of patients regarding SOOCs. For example, patients will be influenced by their objective conditions and the inherent attributes of internet hospitals when choosing an internet clinic, such as patients' disease severity, one-way trip distance to the hospital, the price of the SOOC (the price of specialist outpatient offline consultation is ¥50), the increasing ratio in medical insurance payment for online services compared to offline, and the doctor's recommendation rate (Table 1). Chinese medical insurance is divided into Urban Employee Basic Medical Insurance (UEBMI), Urban and Rural Residents' Basic Medical Insurance (URRBMI) and other insurance, covering nearly 100% of the population. UEBMI was established for individuals employed in the urban sector. URRBMI was implemented to cover urban residents without formal employment and the large rural population. During the survey, the investigator will explain the meaning of each attribute to the patient in detail. For disease severity, the investigator will assume a disease such as hypertension to the patient as an example. When a follow-up visit for medication is needed, it is a mild condition. When rehabilitation guidance is needed, it is a moderate condition. When unstable blood pressure requires a diagnosis, it is a severe condition. For the doctors' recommendation rate, when the doctor does not inform patients of the SOOC approach, it means the doctor weakly recommends it. When the doctor informs patients about the SOOC approach, it means the doctor generally recommends it. When the doctor suggests that the patient can take SOOC services next time, it means the doctor highly recommends it.

**Table 1:** Attributes and levels.

**Attributes**	**Levels**		
Disease severity	Mild	Moderate	Severe
One way trip distance to hospital (min)	Within 30	30–60	More than 60
The price of SOOC (¥)	30	50	70
The increasing ratio of medical insurance payment for online services compared to offline	Invariant	Increase by 10%	Increase by 20%
The convenience of applying SOOC services	Inconvenient	Neutral	Convenient
Doctors' recommendation rate	Weakly recommend	Recommend	Highly recommend

### Orthogonal experimental design

This study used an orthogonal experiment to assist the DCE design in determining the optional attribute combination. The basis of this experiment is to determine the attributes and levels, then combine distinct levels and attributes and finally confirm the optional subject combinations that patients select. Generally, this experiment is critical when many optional subject combinations are derived from a large number of attributes and the corresponding level of attributes, which will increase the difficulty of the DCE design. Therefore, in the actual operation process, the orthogonal design method is often used to determine the optional subject combination and select the representative optional subject combinations for the experimental operation to grasp the overall situation. In addition, of all the final optional subject combinations, the appropriate one should be selected as the control group, and the rest should be paired with it to form different selection sets. In this study, 18 groups of optional subject combinations were determined, and the 9th group was selected as the control group. The rest of the optional subject combinations were paired with the 9th group, forming 17 groups of selection sets (Table 2).

**Table 2:** The optional subject combinations.

**Number**	**Disease severity**	**One way trip distance to hospital (min)**	**The price of SOOC (¥)**	**The increasing ratio of medical insurance payment for online services compared to offline**	**The convenience of applying SOOC services**	**Doctors' recommendation rate**
1	Mild	Within 30	30	Invariant	Inconvenient	Recommend
2	Mild	Within 30	50	Increase by 10%	Convenient	Highly recommend
3	Mild	30–60	30	Increase by 20%	Convenient	Weakly recommend
4	Mild	30–60	70	Invariant	Neutral	Highly recommend
5	Mild	More than 60	50	Increase by 20%	Neutral	Recommend
6	Mild	More than 60	70	Increase by 10%	Inconvenient	Weakly recommend
7	Moderate	Within 30	30	Increase by 20%	Neutral	Highly recommend
8	Moderate	Within 30	70	Invariant	Convenient	Weakly recommend
9	Moderate	30–60	50	Increase by 10%	Neutral	Weakly recommend
10	Moderate	30–60	70	Increase by 20%	Inconvenient	Recommend
11	Moderate	More than 60	30	Increase by 10%	Convenient	Recommend
12	Moderate	More than 60	50	Invariant	Inconvenient	Highly recommend
13	Severe	Within 30	50	Increase by 20%	Inconvenient	Weakly recommend
14	Severe	Within 30	70	Increase by 10%	Neutral	Recommend
15	Severe	30–60	30	Increase by 10%	Inconvenient	Highly recommend
16	Severe	30–60	50	Invariant	Convenient	Recommend
17	Severe	More than 60	30	Invariant	Neutral	Weakly recommend
18	Severe	More than 60	70	Increase by 20%	Convenient	Highly recommend

### Questionnaire design

The questionnaire used in this study includes two parts. The first is basic information and the demographic information of the respondents. The second is the scenario simulation, in which respondents choose from multiple groups of alternatives provided in a set scenario. This alternative uses the 17 groups of selection sets mentioned earlier obtained through orthogonal experimental design. Respondents must choose between two alternatives in each alternative group (Figure 1).

### Data collection

This study selected three specialized and two general tertiary hospitals in Beijing for investigation. These sample hospitals have already built internet hospitals under government approval. The questionnaire was distributed and recycled mainly through random sampling. First, we chose the chronic disease department in the hospital, such as the Cardiovascular Medicine Clinic, Hypertension Clinic, Endocrine Medicine Clinic, Respiratory Medicine Clinic, etc. Then, a convenience sample of waiting patients in these departments was selected and a paper questionnaire was administered to the respondents to maximize the response rate to improve the effectiveness of the questionnaire. Among the inclusion criteria for patient selection were: firstly, patients who were over 18 years old, had been seen at our hospital, had a better understanding of the consultation process, and could clearly express their personal opinions and views. Secondly, patients who voluntarily participated in this survey and gave informed consent. Thirdly, patients who had previous experience with online consultation. Before administering the survey, we asked patients whether they had used any SOOC services before. For those who answer yes, we invited them to participate in the survey.

Although we randomized the sample as much as possible, the actual survey process is still subject to patient self-selection bias, including sample bias and hypothesis bias. For sample bias, firstly this study took a pre-experiment for early identification of bias. In the formal sampling, a balanced distribution of patients' age, gender, and educational levels were considered. Then, before the survey, we established the survey team. The team members received thorough training from the researcher to explain the investigation background, questionnaire design, and factors that need attention. During the process, trained members verbally presented each attribute and its level to the respondent. They were responsible for monitoring and guiding the process and carefully checking the quality and completeness of the questionnaire after the respondents filled it in.

For hypothesis bias, we assume that patients' choices reflect their true underlying preferences. Two strategies can be used to try to reduce the bias. One approach is for our trained investigators to explain this potential bias to patients and encourage them to consider it more carefully (18). Another approach is to use anonymous surveys to help reduce bias. This is because patients may deliberately misrepresent their true preferences during the actual survey.

In addition, this study used Johnson and Orme's rule of thumb to determine the minimum sample size of the main effect DCE model, which is mainly determined by three factors: the number of DCE questions, the number of options, and the maximum level of attributes (19). The formula is *N*> 500*c*/t^*^α, where 500 is a fixed variable, c represents the maximum number of levels in any attribute, t represents the number of DCE questions in each questionnaire, and α refers to the number of options in each question. This formula has been argued to be reasonable and widely used in studies (20–22). Therefore, according to the above formula, the sample size of patients in this study should be >89 (*t* = 17, α = 2, *c* = 6, *n* > 89), which is considered reasonable. By also considering invalid questionnaires, this study finally determined that at least 120 patients were surveyed.

### Logit regression analysis

The international research based on DCE mainly adopts the random effect logit model, random effect probit model, conditional logit model, and mixed logit model (20). In the analysis of this study, the respondents' choice (option 1 or option 2) is taken as the dependent variable, the attributes included in the study are taken as the independent variables, and the conditional logit model is used for regression analysis of the data. Expressed by a mathematical model, this is:


$$\begin{array}{r} \text{Logit P }(\text{Y})=\alpha {\text{ + β}}_{\text{1}}({\text{X}}_{\text{1a}}-{\text{X}}_{\text{1b}}){\text{ + β}}_{\text{2}}({\text{X}}_{\text{2a}}-{\text{X}}_{\text{2b}})\\ \text{ +}......\text{+ }{\text{β}}_{\text{n}}({\text{X}}_{\text{na}}-{\text{X}}_{\text{nb}})\text{ + βX + }\varepsilon \end{array}$$

In the above formula, Y is the result variable, *Y* = 1 represents option 1, and *Y* = 0 represents option 2. α is a constant term. *β*_1_......*β*_*n*_ is the regression coefficient, which reflects the direction and size of this factor's influence on WTP. X refers to other demographic factors in addition to DCE research factors, such as gender, age, and education level. ε is the error term.

After obtaining the regression coefficient, we also calculate the ratio of the coefficient of the nonprice attribute to the coefficient of price and obtain the monetary value of the respondent to other attributes, that is, WTP, as shown in the following formula (23). Amid the formula, *β*_*X*_ stands for nonprice attributes, and *β*_*Price*_ stands for price attributes.


$$\text{WTP }(\text{x})\text{=}\frac{{\text{β}}_{\text{x}}}{{\text{β}}_{\text{Price}}}$$

As SPSS 26.0 software cannot directly realize the conditional logit regression of matched data, it needs a Cox regression model for fixing this problem. If this option is selected, the survival state is 1, so a new list of survival Time variables is added to the data. See the above data entry example. Therefore, this study mainly used conditional logit regression under Cox regression for statistical analysis, and the difference was statistically significant (*P* < 0.05).

## Results

### Basic information about the investigated patients

In this study, 162 valid questionnaires were collected from patients whose ages were in accordance with the normal distribution, with an average of 40.91 ± 15.043 years old. The gender ratio was relatively balanced, with women accounting for 56.8% (*n* = 92). A total of 89.5% of the respondents lived in Beijing, and 74.4% were located in the urban area of Beijing. Regarding the types of medical insurance, UEBMI accounted for 64.2% and URRBMI accounted for 21.6%. In terms of education level, 78.0% of the respondents had a bachelor's degree or above, and 16.7% had a graduate degree or above. Regarding the occupation distribution, all occupations in the questionnaire were represented, with 63.0% of the participants being employees in enterprises and institutions. The monthly income distribution of the participants was relatively balanced, with 45.7% earning <8,000 yuan, and 54.3% earning more than 8,000 yuan; of this group, the monthly income of 39.5% was more than 10,000 yuan. Regarding the round-trip time to go to an offline specialist consultation, 65.4% of the participants spent more than 1 h. Additionally, 30.1% had chronic diseases, and 80% of them were middle-aged and elderly (>40 years old) people. In terms of their health level, 87.7% rated their health status as average or good. From the perspective of cognition and attitude of SOOCs, only 7.4% of people think that the quality of internet hospitals is better than that of offline specialist consultations, and 1.9% of people think that SOOCs are more accurate than offline specialist consultations. However, most people think that the doctors' attitudes and the degree of simplicity of the SOOCs process are the same as or higher than offline specialist consultations, accounting for 85.2 and 83.9%, respectively. A total of 82.7% of the respondents expressed a willingness to try or continue using the SOOC services (Table 3).

**Table 3:** Characteristics of respondents.

**Characteristic**	***n***	**Percentage (%)**
**Gender**		
Male	70	43.2
Female	92	56.8
**Age**		
≤ 40 years old	86	53.1
>40 years old	76	46.9
**Living area**		
Urban area of Beijing	121	74.7
Rural area of Beijing	24	14.8
Other cities	17	10.5
**Medical insurance type**		
UEBMI	104	64.2
URRBMI	35	21.6
Others	23	14.2
**Educational level**		
Junior and below	11	6.8
High school/technical secondary school/vocational high school	23	14.2
Junior college/undergraduate	101	62.3
Graduate student or above	27	16.7
**Occupation**		
Staff members of state organ and public institution	41	25.3
Enterprise/company personnel	61	37.7
Individual household	4	2.5
Retiree	26	16.0
Freelancer	12	7.4
Student	18	11.1
**Monthly income (**¥**)**		
≤ 2,000	17	10.5
2,001–5,000	24	14.8
5,001–8,000	33	20.4
8,001–10,000	24	14.8
10,001–15,000	42	25.9
≥15,001	22	13.6
**Round-trip time for offline specialist consultations**		
Within 0.5 h	13	8.0
0.5–1 h	43	26.5
1–2 h	72	44.5
More than 2 h	34	21.0
Yes	50	30.9
No	112	69.1
**Evaluation of health status by ranking**		
Very poor	13	8.0
Poor	7	4.3
General	75	46.3
Good	50	30.9
Very good	17	10.5
**Service quality in SOOCs vs. offline specialist consultations**		
Lower than offline	89	54.9
Same as offline	61	37.7
Higher than offline	12	7.4
**Accuracy in SOOCs vs. offline specialist consultations**		
Lower than offline	104	64.2
Same as offline	55	34.0
Higher than offline	3	1.9
**Service attitude in SOOCs vs. offline specialist consultations**		
Lower than offline	24	14.8
Same as offline	80	49.4
Higher than offline	58	35.8
**Accessibility in SOOCs vs. offline specialist consultations**		
Lower than offline	26	16.0
Same as offline	37	22.8
Higher than offline	99	61.1
**Willingness to try or continue to use the SOOCs**		
Yes	134	82.7
No	28	17.3

### Analysis of influencing factors of patients' SOOCs

In this study, conditional logit regression was carried out with the respondents' choice of the option (assignment: no = 0, yes = 1) as the dependent variable and six essential attributes as the independent variables. The statistical results show that at least one level of other attributes has a statistically significant impact (*P* < 0.05). For example, the price of SOOC, disease severity, the increasing ratio of medical insurance payments for online services compared to offline, the convenience level of accessing SOOC services, and doctors' recommendation rate have statistically significant effects on patients' choice of medical treatment. From the regression coefficient, patients' degree of preference for SOOCs from high to low is as follows: doctors' recommendation rate (*β*_*highly recommend*_ = 0.999), the convenience level of accessing SOOC services (*β*_*Convenient*_ = 0.760), the increasing ratio in medical insurance payment for online services compared to offline (*β*_*Increase by* 10%_ = 0.545), and disease severity (*β*_*severe*_ = −3.024). The data show that patients are more inclined to choose the SOOC that doctors highly recommend. SOOCs are considered easy to learn and use, and the increasing ratio of medical insurance payment for online services compared with offline is increased by 10%. The disease severity is lighter than these attributes (Table 4).

**Table 4:** Patients' preference for SOOC.

**Attribute and level**	**β**	***S.E***	***Wald***	***P***	***Exp(β)***	**95%** ***CI***	
**The price of SOOC**	−0.027	0.003	90.833	**< 0.001**	0.973	0.968	0.979
**Disease severity** **(Mild group is control)**			562.673	**< 0.001**			
Moderate	−1.021	0.099	106.979	**< 0.001**	0.360	0.297	0.437
Severe	**−3.024**	0.128	559.963	**< 0.001**	0.049	0.038	0.062
**One way trip distance to hospital** **(Within 30 min group is control)**			1.415	0.493			
30–60 min	−0.072	0.119	0.366	0.545	0.930	0.736	1.176
More than 60 min	**−0.140**	0.119	1.385	0.239	0.870	0.689	1.097
**The increasing ratio of medical insurance payment for online services compared to offline** **(invariant group is control)**			32.412	**< 0.001**			
Increase by 10%	**0.583**	0.111	27.407	**< 0.001**	1.791	1.440	2.228
Increase by 20%	0.538	0.117	21.021	**< 0.001**	1.713	1.361	2.156
**The convenience of applying SOOC services (Inconvenient group is control)**			50.529	**< 0.001**			
Neutral	0.540	0.102	28.08	**< 0.001**	1.716	1.406	2.096
Convenient	**0.760**	0.114	44.603	**< 0.001**	2.139	1.711	2.674
**Doctors' recommendation rate (Weakly recommend group is control)**			113.472	**< 0.001**			
Recommend	−0.087	0.113	0.602	0.438	0.916	0.735	1.143
Highly recommend	**0.999**	0.116	74.578	**< 0.001**	2.717	2.165	3.409

The bold values in the table represent statistically significant attributes and their levels, and serve to highlight them.

### Analysis of WTP

**Figure 2:**
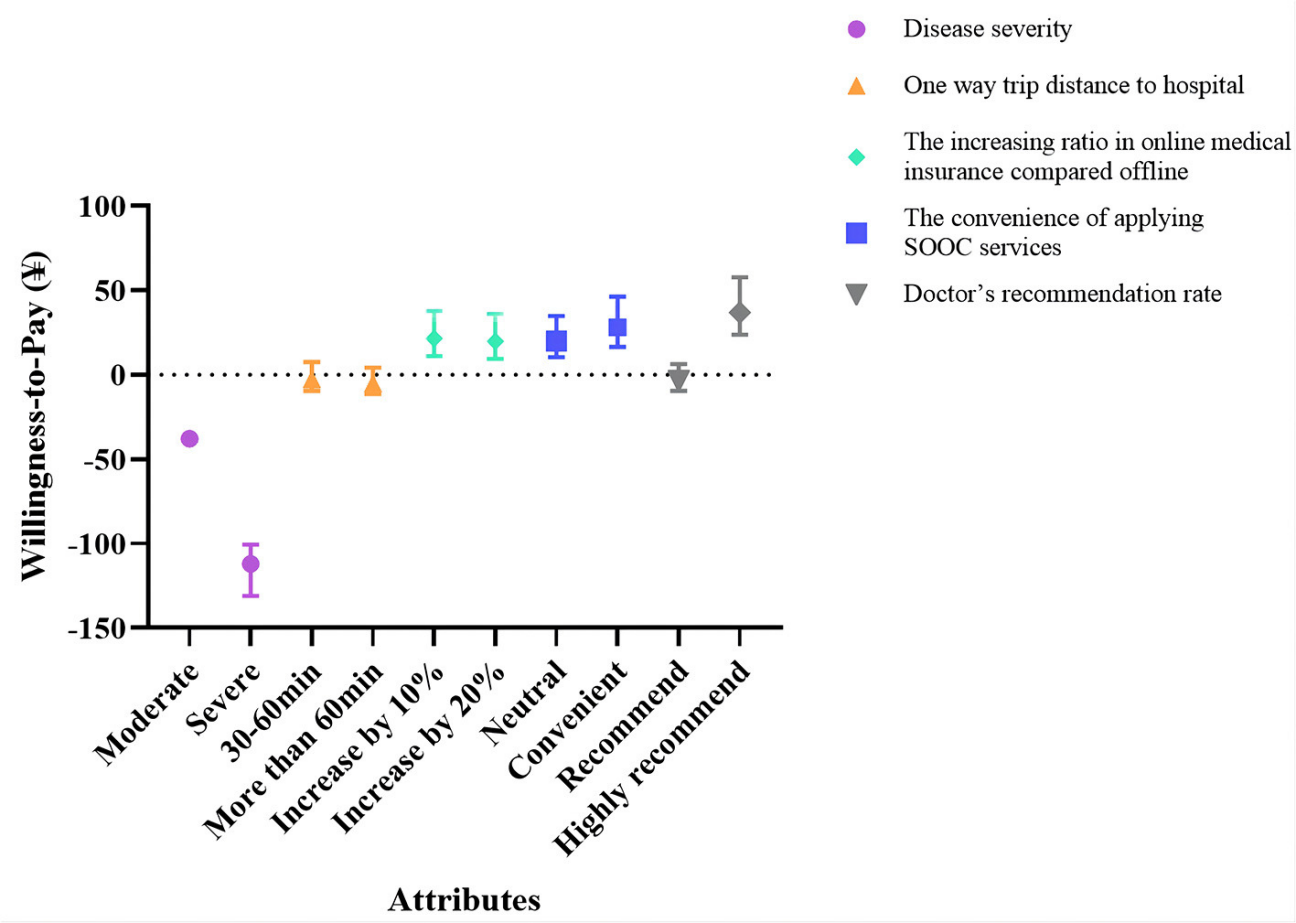
WTP estimates for SOOC attributes.

When there is a price attribute (the price of SOOC) in the setting attribute, researchers can calculate the monetary value of other attributes, that is, WTP, through the model. The positive sign indicates the cost that the patient is willing to sacrifice to obtain the level of a certain attribute, and the negative sign indicates the compensation that should be given to make the patient accept the level of a particular attribute (15, 16). This study estimated the WTP and its 95% confidence interval based on the conditional logit model and used the results to analyze the preference degree and relative value of nonprice attributes of patients. The monetary value evaluation results of each attribute show that patients' WTP for disease severity is higher than other attributes (Figure 2).

Patients' WTP for each attribute is as follows: compared with mild disease severity, patients' WTP for severe disease severity is the weakest, at ¥ −112.00 (95% *CI*: −131.02 to −100.55); compared with the increasing ratio in medical insurance payment for online services compared to offline, patients' WTP for a 10% increase in medical insurance reimbursement is positive, that is, when the ratio increases by 10%, patients are WTP ¥ 21.59 higher (95% *CI*: 11.21–37.75); when the convenience level of applying SOOC services changes from inconvenient to convenient, patients' WTP is ¥ 28.15 higher (95% *CI*: 16.51–46.34); and when the doctors' recommendation rate changes from recommendation to a high recommendation, patients' WTP is ¥ 37.00 higher (95% *CI*: 23.75–57.79) (Table 5).

**Table 5:** WTP estimates for SOOC attributes.

**Attribute and level**	**WTP (¥)**	**95%CI of WTP (**¥**)**	
**The price of SOOC**	_	_	_
**Disease severity (Mild group is control)**			
Moderate	−37.81	−37.33	−39.00
Severe	−112.00	−100.55	−131.02
**One-way trip distance to hospital (Within 30 min group is control)**			
30–60 min	−2.67	7.64	−9.42
More than 60 min	−5.19	4.36	−11.45
**The increasing ratio of medical insurance payment for online services compared to offline (invariant group is**			
**control)**			
Increase by 10%	21.59	37.75	11.21
Increase by 20%	19.93	36.2	9.48
**The convenience of applying SOOC services (Inconvenient group is control)**			
Neutral	20.00	34.87	10.48
Convenient	28.15	46.34	16.51
**Doctors' recommendation rate (Weakly recommend group is control)**			
Recommend	−3.22	6.30	−9.47
Highly recommend	37.00	57.79	23.75

### Changes in the probability of SOOC

**Figure 3:**
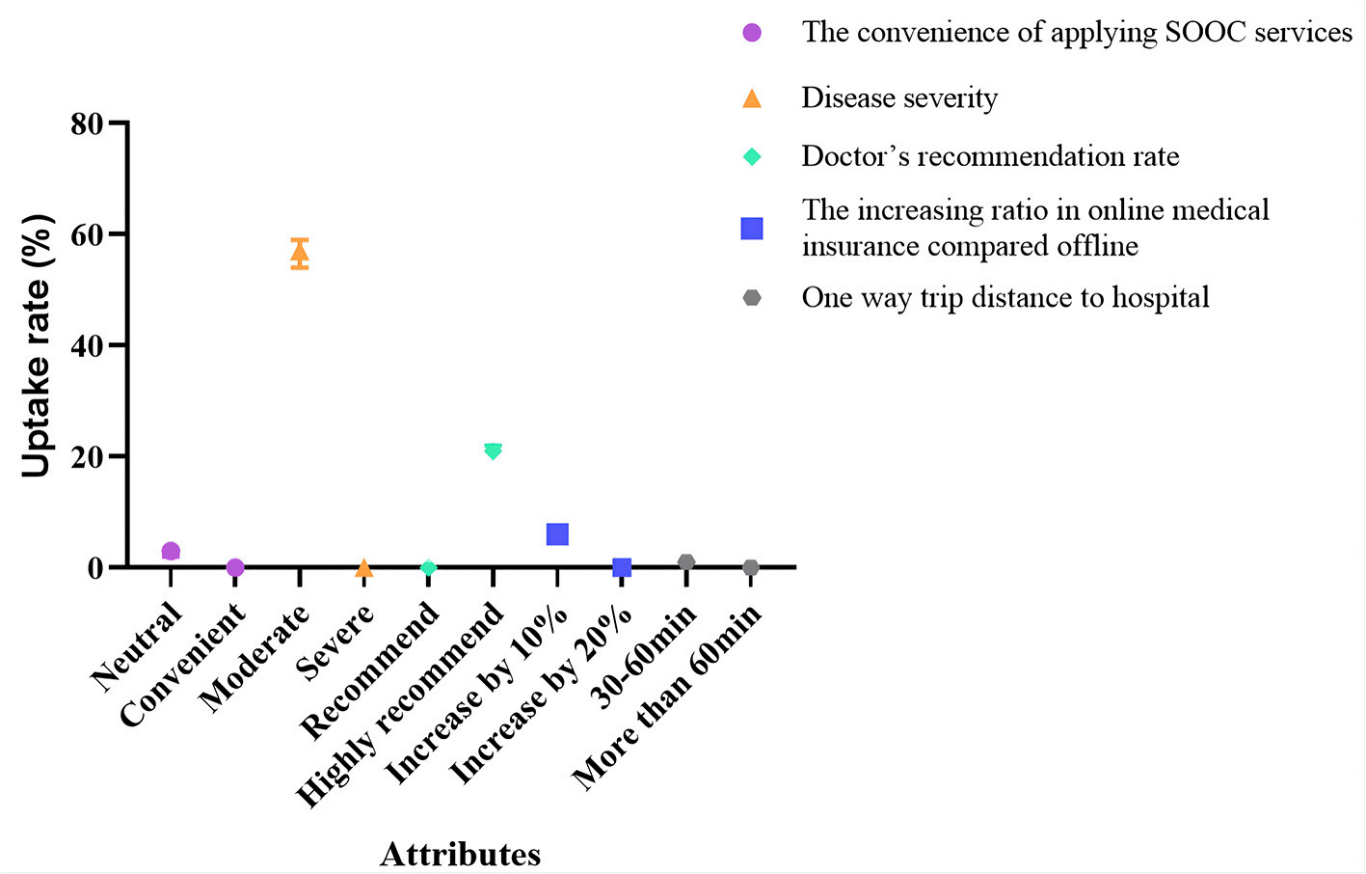
Changes in the probability of SOOC.

With the change in the SOOC attributes, the probability of the service selected is also different. This study found that disease severity and the doctors' recommendation rate were the major factors that influenced respondents' selection of online consultations. For instance, under the circumstance of the two assumed scenarios, the other conditions remained the same. However, the disease was wilder, and the patients were more likely to choose online consultations. In another circumstance of the doctor's recommendation rate, when the recommendation rate varies from low to high, the probability of the patients choosing online consultations increases by 21.0%. This study also shows that when the other properties (i.e., the increasing ratio of medical insurance payment for online services compared to offline, the convenience level of accessing SOOC services, and one-way trip distance to hospital) change, the selection rate changes by no more than 10.0%. They are 6.0, 3.0, and 1.0%, respectively (Figure 3).

### Analysis of each subgroup's choice preference and WTP

This study was divided into subgroups according to age, sex, whether patients had chronic diseases, place of residence, offline round-trip time, and medical insurance type. Then, the preference of patients in subgroups for medical treatment was analyzed. In this study, the price of the SOOC services is set as a continuous variable, and other factors are classified variables. The conditional logit model is used for regression analysis to calculate the WTP.

### Subgroup analysis of people of different ages

A total of 162 people were included in this study, of which 86 were under 40 years old, and 76 were over 40 years old. The results of the conditional logit model analysis (Table 6) show that at least one level of all the attributes of patients under 40 years of age included in the study has statistical significance on their choice preference. However, the attribute of medical distance of patients over 40 years of age has no statistical significance, and at least one level of other attributes has statistical significance. The statistical results show that the WTP of patients in the low-age group is higher than that of patients in the high-age group for all attribute levels. Specifically, in terms of disease severity, in the high-age group, the WTP of patients with severe disease is ¥ −87.13 (95% *CI*: −105.81 to −78.92) compared with that of people with mild diseases, while that of patients in the low-age group is only ¥ −141.71 (95% *CI*: −188.26 to −119.79). When the increasing ratio of medical insurance payment for online services compared to offline is increased by 20%, the WTP of patients in the low-age group is WTP ¥ 29.50 higher (95% *CI*: 11.78–64.80), while that of those in the high-age group is only WTP ¥ 12.61 (95% *CI*: 1.75–32.09). In terms of the convenience level of applying SOOC services, when it changed from inconvenient to convenient, patients in the low-age group were willing to pay ¥ 33.75 more (95% *CI*: 15.73–69.74), while patients in the high-age group were only willing to pay ¥ 24.00 (95% *CI*: 10.91–47.89). As for the doctor's recommendation, when it changed from recommended to highly recommended, patients in the low-age group were willing to pay ¥ 51.25 more (95% *CI*: 28.51–96.82), while those in the high-age group were only willing to pay ¥ 26.26 (95% *CI*: 12.79–50.92).

**Table 6:** Conditional logit regression analysis and the WTP for patients of different ages.

**Attribute and level**	≤ **40 years old**				**>40 years old**			

	β	**WTP (**¥**)**	**95%*****CI*** **of WTP (**¥**)**		β	**WTP (**¥**)**	**95%*****CI*** **of WTP (**¥**)**	
**The price of SOOC**	−0.024^***^	_	_	_	−0.031^***^	_	_	_
**Disease severity**								
**(Mild group is control)**								
Moderate	−1.199^***^	−49.96	−56.96	−46.95	−0.861^***^	−27.77	−26.47	−29.25
Severe	−3.401^***^	−141.71	−188.26	−119.79	−2.701^***^	−87.13	−105.81	−78.92
**One-way trip distance to hospital**								
**(Within 30 min group is control)**								
30–60 min	−0.239	−9.96	−18.53	6.53	0.074	2.39	−6.55	18.05
More than 60 min	−0.448^**^	−18.67	−6.60	−25.08	0.138	4.45	−34.90	20.90
**The increasing ratio of medical insurance payment for online services compared to offline (invariant group is control)**								
Increase by 10%	0.668^***^	27.83	10.73	61.81	0.556^***^	17.94	6.63	38.48
Increase by 20%	0.708^***^	29.50	11.78	64.80	0.391^**^	12.61	1.75	32.09
**The convenience of applying SOOC services (Inconvenient group is control)**								
Neutral	0.55^***^	22.92	8.53	51.61	0.541^***^	17.45	6.55	37.21
Convenient	0.81^***^	33.75	15.73	69.74	0.744^***^	24.00	10.91	47.89
**Doctors' recommendation rate (Weakly recommend group is control)**								
Recommend	−0.084	−3.50	−12.48	14.03	−0.113	−3.65	−11.16	9.31
Highly recommend	1.23^***^	51.25	28.51	96.82	0.814^***^	26.26	12.79	50.92

Standardized coefficients are shown with the associated statistics in parentheses.

*** , and

** denote significance levels at 1 and 5%, respectively.

### Subgroup analysis of people with different chronic diseases

A total of 162 people were included in this study, including 50 patients with chronic diseases and 112 patients with nonchronic diseases. The results of the conditional logit model analysis (Table 7) show that there is no statistical significance in the two groups except for the attribute of medical distance, and at least one level of other attributes has statistical significance on the choice preference. The statistical results show that the WTP of nonchronic patients is higher than that of chronic patients. Specifically, in terms of disease severity, compared with those with mild diseases, the WTP for those with severe disease in the nonchronic disease group was ¥ −118.16 (95% *CI*: −146.40 to −103.02), while the WTP for the chronic disease group was slightly higher at ¥ −106.48 (95% *CI: –*131.78 to –91.17); When the increasing ratio of medical insurance payment for online services compared with offline was increased by 20%, the WTP of patients with chronic diseases was WTP ¥ 24.39 higher (95% *CI*: 7.415–6.61), while that of patients with nonchronic diseases was only ¥ 18.16 (95% *CI*: 5.79–39.89). Regarding the convenience level of accessing SOOC services, when it changed from being inconvenient to convenient, the WTP of patients with chronic diseases was ¥ 22.16 more (95% *CI*: 6.21–52.47), while that of patients with nonchronic diseases was WTP ¥ 31.20 (95% *CI*: 16.40–57.47), which was higher than those with chronic diseases. Regarding the doctor's recommendation rate, when it changed from recommended to highly recommended, the WTP of patients with chronic diseases was ¥ 32.32 more (95% *CI*: 13.63–67.55), while that of patients with nonchronic diseases was WTP ¥ 40.60 more (95% *CI*: 23.70–70.66).

**Table 7:** Conditional logit regression analysis and the WTP based on the factor that whether the patients have chronic diseases.

**Attribute and level**	**Patients with chronic diseases**				**Patients with non-chronic diseases**			

	β	**WTP (**¥**)**	**95%*****CI*** **of WTP (**¥**)**		β	**WTP (**¥**)**	**95%*****CI*** **of WTP (**¥**)**	
**The price of SOOC**	−0.031^***^	_	_	_	−0.025^***^	_	_	_
**Disease severity**								
**(Mild group is control)**								
Moderate	−0.997^***^	−32.16	−30.63	−32.11	−1.048^***^	−41.92	−44.70	−40.77
Severe	−3.301^***^	−106.48	−131.78	−91.17	−2.954^***^	−118.16	−146.40	−103.02
**One-way trip distance to hospital (Within 30 min group is control)**								
30–60 min	−0.129	−4.16	−13.81	15.11	−0.043	−1.72	−10.12	12.90
More than 60 min	−0.143	−4.61	−14.11	14.35	−0.141	−5.64	−13.19	7.41
**The increasing ratio of medical insurance payment for online services compared to offline (invariant group is control)**								
Increase by 10%	0.980^***^	31.61	13.20	66.3	0.430^**^	17.20	5.50	37.8
Increase by 20%	0.756^**^	24.39	7.41	56.61	0.454^**^	18.16	5.79	39.89
**The convenience of applying SOOC services (Inconvenient group is control)**								
Neutral	0.625^**^	20.16	6.03	47.03	0.501^***^	20.04	8.33	40.69
Convenient	0.687^**^	22.16	6.21	52.47	0.780^***^	31.20	16.40	57.47
**Doctors' recommendation rate (Weakly recommend group is control)**								
Recommend	−0.234	−7.55	−15.71	8.90	−0.030	−1.20	−9.26	12.72
Highly recommend	1.002^***^	32.32	13.63	67.55	1.015^***^	40.60	23.70	70.66

Standardized coefficients are shown with the associated statistics in parentheses.

*** , and

** denote significance levels at 1 and 5%, respectively.

### Subgroup analysis of people with different incomes

A total of 162 people were included in this study, including 74 people in the low-income group (monthly income ≤ 8,000 yuan) and 88 people in the high-income group (monthly income >8,000 yuan). The results of the conditional logit model analysis (Table 8) show that there is no statistical significance in the two groups except for the attribute of medical distance, and at least one level of other attributes has statistical significance on the choice preference. The statistical results show that the WTP of high-income patients is higher than that of low-income patients. Specifically, in terms of disease severity, compared with the mild group, the WTP for severe diseases in the low-income group was ¥ –78.40 (95% *CI: –*70.56 to –87.17), while that in the high-income group was ¥ –164.15 (95% *CI: –*133.34 to –243.32). When the increasing ratio of medical insurance payment for online services compared to offline is increased by 20%, the WTP of the high-income group is ¥ 23.90 more (95% *CI*: 6.05–65.57), while that of the low-income group is slightly lower at ¥ 17.86 (95% *CI*:6.40–35.36). Regarding the convenience level of applying SOOC services, when it changed from inconvenient to convenient, high-income patients' WTP was ¥ 33.85 more (95% *CI*: 13.60–81.25), while low-income patients were only WTP ¥ 24.94 (95% *CI*: 12.31–43.99). Regarding the doctor's recommendation rate, when it changed from a recommendation to a high recommendation, patients in the high-income group were willing to pay ¥ 49.30 more (95% *CI*: 24.75–107.22), while patients in the low-income group were only willing to pay ¥ 29.77 (95% *CI*: 16.00–50.46).

**Table 8:** Conditional logit regression analysis and the WTP of patients with different income conditions.

**Attribute and level**	**Low-income group**				**High-income group**			

	β	**WTP (**¥**)**	**95%*****CI*** **of WTP (**¥**)**		β	**WTP (**¥**)**	**95%*****CI*** **of WTP (**¥**)**	
**The price of SOOC**	−0.035^***^	_	_	_	−0.02^***^	_	_	_
**Disease severity**								
**(Mild group is control)**								
Moderate	−0.877^***^	−25.06	−26.57	−21.45	−1.144^***^	−57.2	−72.85	−51.39
Severe	−2.744^***^	−78.40	−87.17	−70.56	−3.283^***^	−164.15	−243.32	−133.34
**One-way trip distance to hospital (Within 30 min group is control)**								
30–60 min	−0.130	−3.71	−11.02	8.15	−0.038	−1.9	−12.93	23.00
More than 60 min	−0.230	−6.57	−13.07	4.24	−0.084	−4.2	−14.69	19.47
**The increasing ratio of medical insurance payment for online services compared to offline (invariant group is control)**								
Increase by 10%	0.571^***^	16.31	5.83	32.33	0.602^***^	30.1	10.77	75.33
Increase by 20%	0.625^***^	17.86	6.40	35.36	0.478^**^	23.9	6.05	65.57
**The convenience of applying SOOC services (Inconvenient group is control)**								
Neutral	0.715^***^	20.43	9.51	36.99	0.410^**^	20.5	4.98	56.71
Convenient	0.873^***^	24.94	12.31	43.99	0.677^***^	33.85	13.60	81.25
**Doctors' recommendation rate (Weakly recommend group is control)**								
Recommend	−0.100	−2.86	−9.73	8.30	−0.084	−4.2	−14.09	18.08
Highly recommend	1.042^***^	29.77	16.00	50.46	0.986^***^	49.3	24.75	107.22

Standardized coefficients are shown with the associated statistics in parentheses.

*** , and

** denote significance levels at 1 and 5%, respectively.

### Subgroup analysis of people with different medical insurance types

A total of 162 people were included in this study, including 104 patients with UEBMI, 35 patients with URRBMI, and 23 patients with other medical insurance (such as FMC). The results of the conditional logit model analysis (Table 9) show that there is no statistical significance among the three groups of patients, except for the attribute of medical distance, and at least one level of other attributes has statistical significance on their choice preference. The statistical results show that patients with different medical insurance types are WTP for different attributes. The details are as follows: in terms of disease severity, compared with mild cases, the WTP for severe diseases in the URRBMI group was ¥ −81.36 (95% *CI: –*102.54 to −72.16), while the WTP for patients in the other medical insurances group was ¥ −115.39 (95% *CI: –*208.18 to −90.17), and that of URBMI group was ¥ −122.24 (95% *CI: –*151.34 to −107.38). When the increasing ratio of medical insurance payment for online services compared to offline is increased by 20%, the other medical insurances group has the highest WTP, which is ¥ 31.18 (95% *CI*: 5.31–125.34), while the URRBMI group is slightly lower at ¥ 24.14 (95% *CI*: 6.97–60.24), and UEBMI group is ¥ 15.60 (95% *CI*: 3.40–37.05). Regarding the convenience of learning and using online medical treatment, when it changed from inconvenient to convenient, the URRBMI group was WTP an extra ¥ 33.81 (95% *CI*: 14.77–74.08), and the third group of patients was WTP an extra ¥ 31.11 (95% *CI*: 6.13–122.03). Regarding the doctor's recommendation rate, when it changed from a recommendation to a high recommendation, the third group was WTP ¥ 47.68 (95% *CI*: 15.83–163.52) more, while the UEBMI group was WTP ¥ 37.24 (95% *CI*: 20.71–66.57), while the URRBMI group was willing to pay the lowest amount.

**Table 9:** Conditional logit regression analysis and the WTP of patients with different medical insurance types.

**Attribute and level**	**Urban Employee Basic Medical Insurance**				**Urban and Rural Residents' Basic Medical Insurance**				**Other medical insurances**			

	β	**WTP** **(**¥**)**	**95%*****CI*** **of WTP (**¥**)**		β	**WTP (**¥**)**	**95%*****CI*** **of WTP (**¥**)**		β	**WTP (**¥**)**	**95%*****CI*** **of WTP (**¥**)**	
**The price of SOOC**	−0.025^***^	_	_	_	−0.036^***^	_	_	_	−0.028^**^	_	_	_
**Disease severity**												
**Mild group is control)**												
Moderate	−1.057^***^	−42.28	−45.07	−41.11	−0.874^***^	−24.28	−18.51	−27.35	−1.061^***^	−37.89	−43.85	−36.28
Severe	−3.056^***^	−122.24	−151.34	−107.38	−2.929^***^	−81.36	−102.54	−72.16	−3.231^***^	−115.39	−208.18	−90.17
**One way trip distance to hospital**												
**Within 30 min group is control)**												
30–60 min	−0.033	−1.32	−10.08	13.89	−0.302	−8.39	−17.63	10.51	−0.023	−0.82	−16.28	55.57
More than 60 min	−0.141	−5.64	−13.58	8.08	−0.193	−5.36	−15.29	15.07	−0.119	−4.25	−17.08	42.58
**The increasing ratio of medical insurance payment for online services compared to offline (invariant group is control)**												
Increase by 10%	0.579^***^	23.16	9.76	46.87	0.739^***^	20.53	5.37	52.44	0.387	13.82	−4.79	81.53
Increase by 20%	0.390^**^	15.60	3.40	37.05	0.869^***^	24.14	6.97	60.24	0.873^**^	31.18	5.31	125.34
**The convenience of applying SOOC services (Inconvenient group is control)**												
Neutral	0.353^**^	14.12	3.37	33.00	1.079^***^	29.97	13.02	65.8	0.647^**^	23.11	2	99.84
Convenient	0.594^***^	23.76	10.07	47.97	1.217^***^	33.81	14.77	74.08	0.871^**^	31.11	6.13	122.03
**Doctors' recommendation rate (Weakly recommend group is control)**												
Recommend	−0.069	−2.76	−10.88	11.26	−0.493^**^	−13.69	−20.49	0.00	0.441^***^	15.75	−4.54	89.52
Highly recommend	0.931^***^	37.24	20.71	66.57	1.055^***^	29.31	11.02	67.91	1.335	47.68	15.83	163.52

Standardized coefficients are shown with the associated statistics in parentheses.

*** , and

** denote significance levels at 1 and 5%, respectively.

### Subgroup analysis of people with different distance

A total of 162 people were included in this study, including 56 patients in the short-distance group (the time to offline hospital <1 h), and 106 patients in the long-distance group (the time to offline hospital ≥1 h). The results of the conditional logit model analysis (Table 10) show that there is no statistical significance among the two groups of patients, except for the attribute of one-way trip distance to the hospital, and at least one level of other attributes has statistical significance on their choice preference. The WTP in the short-distance group was higher than that in the long-distance group. The details are as follows: when the increasing ratio of medical insurance payment for online services compared to offline is increased by 20%, the short-distance group has the highest WTP, which is ¥ 24.08 (95% *CI*: 6.64–59.65), while the long-distance group is slightly lower at ¥ 17.89 (95% *CI*: 6.27–37.00). Regarding the doctor's recommendation rate, when it changed from a general recommendation to a high recommendation, the short-distance group was WTP ¥ 41.73 (95% *CI*: 19.97–86.22) more, while the long-distance group was WTP ¥ 34.82 (95% *CI*: 20.10–59.16).

**Table 10:** Conditional logit regression analysis and the WTP of patients with different distance.

**Attribute and level**	**Short-distance group**				**Long-distance group**			

	β	**WTP (**¥**)**	**95%*****CI*** **of WTP (**¥**)**		β	**WTP (**¥**)**	**95%*****CI*** **of WTP (**¥**)**	
**The price of SOOC**	−0.026^***^	_	_	_	−0.028^***^	_	_	_
**Disease severity**								
**Mild group is control)**								
Moderate	−1.021^***^	−39.27	−40.31	−39.05	−1.028^***^	−36.71	−37.10	−36.70
Severe	−2.895^***^	−111.35	−144.46	−96.10	−3.122^***^	−111.50	−131.78	−99.50
**One-way trip distance to hospital (Within 30 min group is control)**								
30–60 min	−0.356	−13.69	−21.83	2.51	−0.090	3.21	−5.91	18.12
More than 60 min	−0.341	−13.12	−21.34	3.29	−0.026	−0.93	−9.10	12.40
**The increasing ratio of medical insurance payment for online services compared to offline (invariant group is control)**								
Increase by 10%	0.799^***^	30.73	12.26	68.53	0.462^**^	16.50	5.56	34.48
Increase by 20%	0.626^**^	24.08	6.64	59.65	0.501^**^	17.89	6.27	37.00
**The convenience of applying SOOC services (Inconvenient group is control)**								
Neutral	0.667^***^	25.65	9.52	58.57	0.466^***^	16.64	6.27	33.73
Convenient	0.754^***^	29.00	10.82	66.17	0.772^***^	27.57	14.34	49.45
**Doctors' recommendation rate (Weakly recommend group is control)**								
Recommend	−0.150	−5.77	−14.86	12.50	−0.034	−1.21	−79.10	11.60
Highly recommend	1.085^***^	41.73	19.97	86.22	0.975^***^	34.82	20.10	59.16

Standardized coefficients are shown with the associated statistics in parentheses.

*** , and

** denote significance levels at 1 and 5%, respectively.

## Discussion

The method adopted in this study is the discrete choice experiment (DCE), a stated preference survey. It explores respondents' preferences and WTP by studying how they choose between different attribute levels and price conditions so that decisions can be made directly, rationally, and realistically (16). It has been widely used in health economics and policy research and used to inform the design of health care services (24–26). When designing health service plans and formulating health policies, it is necessary for policy-makers to understand what factors can affect patients' choice preferences for services such as the SOOCs provided by internet hospitals. DCE is an effective way to achieve this.

Initially, this study compared patient perceptions of SOOCs with offline consultations. The results suggest that most patients are willing to use SOOCs. However, they still feel that the quality and accuracy of SOOCs are lower than offline consultations, and there are certain technical risks. The specific outcome is as follows. In terms of user experience, most people think that the doctor's attitudes and the simplicity of the process during SOOCs are the same as or better than those during offline consultations. In terms of service quality, only 7.4% of people think that the quality of SOOCs is higher than that of offline consultations. In terms of technical risks, more than half of patients believe that SOOCs are less accurate than offline consultations. Studies from China and abroad have found that low patient willingness to choose online consultations is associated with patient concerns about the quality of online consultation services, particularly about the accuracy of treatment (27, 28). This may be related to some factors, such as the immature technology of online consultations, insufficient protection of confidential data, and unsound laws and regulations, which make the safety guarantee and liability tracing of SOOCs more difficult than offline consultations.

Then, this study analyzed the factors influencing patient choice preference. The patient's preferences for the attributes, from high to low, are the doctor's recommendation rate (*β*_*highly recommend*_ = 0.999), the convenience level of applying SOOC services (*β*_*Convenient*_ = 0.760), the increasing ratio in medical insurance payment for online services compared to offline (*β*_*Increase by* 10%_ = 0.545), and the disease severity (*β*_*severe*_ = −3.024). The reasons why these four attributes affect their preferences are explained as follows. Doctor-patient information asymmetry is the main reason why a doctor's recommendation rate has a great impact on patient preferences. Usually, patients have insufficient awareness of internet hospitals and their disease conditions, and doctors, as service providers, have professional and comprehensive knowledge about this service (29). Therefore, patients will trust the doctor's point of view, and the doctors' recommendations can definitely influence patients' choices. The second statistically significant factor is the convenience of accessing SOOC services. A study from the United Kingdom found that busy young people had a lower preference for offline consultations and a higher preference for online consultations (30), which was explained by the more attractive convenience of online consultations. The third factor is the increasing ratio of medical insurance payment for online services compared with offline rates, which determines how much patients' economic benefits can be increased. It could also affect patients' acceptance and affordability of SOOCs. The last factor is disease severity. The regression coefficient for this variable is negative, which indicates that disease severity is negatively correlated with patients' choice preferences. Patients with more severe illnesses or injuries are more likely to choose SOOCs for physical examinations and treatments (31), which cannot be met by offline consultations. With the change in the level of attributes, this study also finds that disease severity and doctors' rate of recommendation have the greatest impact on the probability of patients' selecting SOOCs. For instance, when the other attribute levels remain the same, but the disease is milder or the doctor's recommendation rate is higher, patients have a higher probability of choosing a SOOC. Notably, the results of this study showed that the attribute of one-way trip distance to the hospital did not have a significant effect on patient choice preference. This is inconsistent with previous studies. A study from Pakistan suggested that the attribute of distance to hospital may have a significant impact on patients' choice preferences in underdeveloped areas (32). The possible reason was that the number of patients from other provinces or cities who were treated in the hospitals of Beijing decreased due to COVID-19. In our survey, 79% of the respondents were <2 h from the offline hospital. So, the distance factor did not have a significant effect on local patients.

In addition, according to the evaluation results of the monetary value of each attribute, patients' WTP for disease severity is higher than other attributes. Compared with mild disease severity, patients' WTP for severe diseases is the weakest, at ¥ −112.00. This means that if patients suffer from severe diseases, the SOOC will only be selected when they pay ¥ 112.00 less than before. When patients perceive that their diseases are severe, they often have a greater demand for face-to-face communication with doctors and physical examinations. Most of them will undergo emergency and complex treatments, including surgery and hospitalization. These services are not available in the SOOC model. Unlike other factors, disease severity is directly related to the patient's future health, so patients are willing to pay more for quick and reliable treatment. Prior exploration found that patients with mild and moderate diseases would utilize SOOCs more frequently (33).

The conditional logit model further suggested the existence of WTP heterogeneity among patients with different sociodemographic characteristics. First, the WTP of patients in the low-age group was higher than that of patients in the high-age group, which is consistent with the finding of a cross-sectional study from Japan (34). That study indicated that during the COVID-19 pandemic, despite an overall increase in the use of online consultations by the patient group, younger patients were still more likely to use it than older patients. This finding can probably be attributed to the ability of younger patients to accept new things and learn digital technologies, which also reflects a technological gap and a digital divide caused by age (35–37). Second, the WTP of most patients in the high-income group was higher than that of the patients in the low-income group, which echoes the findings of previous research in the United States (38). This is mainly because high-income patients prefer to spend more money for more convenient services and better medical experiences. Third, the findings revealed that patients with different medical insurance types have different WTPs for different attributes. Among them, disease severity has a greater impact on the WTP for patients with UEBMI, and the medical insurance payment for online services reimbursement ratio has a greater impact on the WTP for patients with URRBMI and other types of medical insurance. The reason may be that UEBMI provides patients greater compensation benefits than URRBMI and other medical insurance. Patients with UEBMI are more concerned about whether their disease conditions are suitable for using SOOCs, so they have a higher WTP for severe diseases. Due to the lower compensation levels of URRBMI and other medical insurances, the medical insurance payment for online services reimbursement ratio is a greater concern for patients with these medical insurances; thus, they have a higher WTP for the increasing ratio in medical insurance payment for online services compared with offline services. Finally, this study found that the WTP of patients with nonchronic diseases was basically higher than that of patients with chronic diseases. On the one hand, patients with chronic diseases are mostly older, and older patients have a lower WTP. On the other hand, for patients with chronic diseases, their symptoms may be complex, the treatment cycle may be long, and multiple departments are often involved. To effectively and conveniently handle chronic diseases, internet hospitals that provide SOOCs need to meet various stringent requirements. However, they are not mature or developed enough to meet them currently. The needs of patients with chronic diseases are not being adequately met in SOOCs. Therefore, when the level of most attributes is adjusted, the WTP of patients with chronic diseases is generally lower.

The above analysis provides many directions for developing policy recommendations to improve the SOOC system. On the one hand, as price factors play a significant role in patients' choice preferences, health authorities in China should reasonably price the SOOCs and appropriately increase the medical insurance payment for online services reimbursement ratio for patients who are eligible for online services, such as those with chronic diseases (39). However, since the convenience of accessing SOOC services is widely considered, it should be emphasized in policy and used as an indispensable indicator of service quality. Previous studies confirmed that the perceived ease of use of SOOC services is a determinant of patients' adoption intention toward the online service (40, 41), especially among older adults (31). Accordingly, internet hospitals should be patient-centered, further optimize the service delivery process to improve the ease of using SOOCs and provide patients with multiple convenient and easy-to-use channels to consult doctors, such as internet hospital apps and WeChat official accounts (6, 14). In addition, since doctors' recommendations have a significant impact on patients' choices, publicity and training for doctors should be strengthened so that patients have a better understanding of and trust in the SOOC model, thereby increasing their use of it.

## Limitation

This study contributes to the knowledge of patients' choice preferences for SOOCs. Nonetheless, some limitations should be noted in this study. The first is that this study analyzes the stated preferences of the respondents to hypothetical scenarios, which may not necessarily reflect the choices they would make in a real setting. Consistency between revealed preferences and stated preferences should be explored in future research. The second limitation is that although the sample size in this study met the requirements of the experiment, it still needs to be expanded in future studies. And patients' choice preferences for long distances from the hospital need to be explored in a follow-up study. SOOCs face different situations in different regions. When formulating relevant policies, various practical factors, including social background, need to be comprehensively considered. The generalization of the findings requires further research.

## Conclusion

This study demonstrates that patients' choice preferences and WTP for SOOCs are influenced by a range of attributes. Among them, disease severity and the doctor's recommendation rate have the most significant effect, revealing that appropriate diseases and effective physician-patient interactions are essential for driving the SOOC model. The attributes that influence whether patients are more inclined to choose SOOCs are as follows: doctors' high recommendation, the convenience of accessing SOOC services, when the ratio of medical insurance payment for online services compared with offline is 10% higher, and a mild disease severity. Patients under 40 years old, with high incomes or nonchronic diseases generally have a higher WTP. Based on the findings, patients' preferences and WTP for SOOCs will be enhanced by increasing the medical insurance payment for online services reimbursement ratio, optimizing the service delivery process, developing humanized and convenient systems for patients of advanced age, and training doctors to provide patients with more professional recommendations and interpretations about SOOCs.

## Data availability statement

The raw data supporting the conclusions of this article will be made available by the authors, without undue reservation.

## Ethics statement

The studies involving human participants were reviewed and approved by the Ethics Committee of Capital Medical University (Approval No. Z2022SY024). The patients/participants provided their written informed consent to participate in this study.

## Author contributions

CM and MW contributed to the conception and design of the study. YL and MW designed, performed the investigation, and wrote the first draft of the manuscript. MW participated in the acquisition and analysis of data. YL assisted with data collection. YL and CM revised the manuscript. CM is responsible for the overall management of this study. All authors read and approved the final manuscript.
