# Corrigendum: Patients' choice preferences for specialist outpatient online consultations: a discrete choice experiment

**DOI:** 10.3389/fpubh.2023.1206854

**Published:** 2023-05-10

**Authors:** Mengqiu Wu, Yuhan Li, Chengyu Ma

**Affiliations:** ^1^Key Laboratory of Carcinogenesis and Translational Research (Ministry of Education/Beijing), Laboratory of Genetics, Peking University Cancer Hospital and Institute, Beijing, China; ^2^School of Public Health, Capital Medical University, Beijing, China

**Keywords:** internet hospitals, specialist outpatient online consultations (SOOC), preference, discrete choice experiment, logit regression

In the published article, there was an error in the Funding statement. This study has been funded by the Natural Science Foundation of Beijing Municipality (Grant Number: 9222993). The correct Funding statement appears below.

