# Supplementary material for: Patients' choice preferences for specialist outpatient online consultations: A discrete choice experiment
Source: Front Public Health. 2023 Jan 5;10:1075146. doi: 10.3389/fpubh.2022.1075146 (PMC9850164; doi:10.3389/fpubh.2022.1075146)
Supplement: Supplementary file 1 [file Data_Sheet_1.docx]

**Supplement 1：Patient's Basic Information**

*Instructions: Please answer the following questions to the best of your ability.*

1. Gender:□Male □Female
2. Age:_________________
3. Where you live:□Urban area of Beijing□Rural area of Beijing□Other cities
4. Your type of health insurance:

□Urban Employee Basic Medical Insurance (UEBMI)

□Urban and Rural Residents’ Basic Medical Insurance (URRBMI)

□Others_________________

5. Your education level:

□Junior and below

□High school / Technical secondary school / Vocational high school

□Junior college/ Undergraduate

□Graduate student or above

6. Your occupation:

□Staff members of state organs and public institution

□Enterprise/Company Personnel

□Individual household

□Retiree

□Freelancer

□Student

7. Your monthly income (¥):

□≤2000

□2001-5000

□5001-8000

□8001-10000

□10001-15000

□≥15001

8. The round-trip time for your **offline specialist consultations** is usually:

□Within 0.5 hours

□0.5 hours to 1 hour

□1 hour to 2 hours

□More than 2 hours

1. Whether you have chronic diseases?

□Yes

□No

10. How would you evaluate your health status by ranking :

□Very poor

□Poor

□General

□Good

□Very good

11. What do you think is the difference between the current **service** **quality** of SOOCs and offline **specialist** consultations?

□ Lower than offline

□ Same as offline

□ Higher than offline

11.1 What do you think is the difference between the **accuracy** of SOOCs and offline **specialist** consultations?

□ Lower than offline

□ Same as offline

□ Higher than offline

11.2 What do you think is the difference between the **doctors’ attitude** of SOOCs and offline **specialist** consultations?

□ Lower than offline

□ Same as offline

□ Higher than offline

11.3 What do you think is the difference between the **accessibility** of SOOCs and offline **specialist** consultations?

□ Lower than offline

□ Same as offline

□ Higher than offline

12. Are you willing to try or continue to use SOOCs?

□Yes□ No

**Supplement 2：Medical Treatment Choice Set**

*Instructions:*

- ***Imagine that when you need to seek a medical treatment, you may be faced with two different scenarios, namely option A and option B as below, which differ in six attributes.***
- ***In each of the following choice sets, you need to make comprehensive consideration and balance according to your own preferences, and choose one of the two options for which you would prefer to take SOOC.***
- ***At present, it is known that the price of*** specialist outpatient offline consultation ***is 50 yuan.***

**Choice Set 1:** Which option would you prefer from the following choice? Please tick your choice.

| Attributes | Option A | Option B |
| --- | --- | --- |
| Disease severity | Medium | **Mild** |
| One way trip distance to hospital (min) | 30-60 | **Within 30** |
| The price of SOOC(¥) | 50 | **30** |
| The increasing ratio of online medical insurance compared to offline | Increase by 10% | **Invariant** |
| The convenience of applying SOOC services | Neutral | **Inconvenient** |
| Doctors’ recommendation rate | Weakly recommend | **Recommend** |
|  | □ | □ |

**Choice Set 2:** Which option would you prefer from the following choice? Please tick your choice.

| Attributes | Option A | Option B |
| --- | --- | --- |
| Disease severity | Medium | **Mild** |
| One way trip distance to hospital (min) | 30-60 | **Within 30** |
| The price of SOOC(¥) | 50 | 50 |
| The increasing ratio of online medical insurance compared to offline | Increase by 10% | Increase by 10% |
| The convenience of applying SOOC services | Neutral | **Convenient** |
| Doctors’ recommendation rate | Weakly  recommend | **Highly**  **recommend** |
|  | □ | □ |

**Choice Set 3：**Which option would you prefer from the following choice? Please tick your choice.

| Attributes | Option A | Option B |
| --- | --- | --- |
| Disease severity | Medium | **Mild** |
| One way trip distance to hospital (min) | 30-60 | 30-60 |
| The price of SOOC(¥) | 50 | 30 |
| The increasing ratio of online medical insurance compared to offline | Increase by 10% | Increase by 20% |
| The convenience of applying SOOC services | Neutral | **Convenient** |
| Doctors’ recommendation rate | Weakly  recommend | Weakly  recommend |
|  | □ | □ |

**Choice Set 4：**Which option would you prefer from the following choice? Please tick your choice.

| Attributes | Option A | Option B |
| --- | --- | --- |
| Disease severity | Medium | **Mild** |
| One way trip distance to hospital (min) | 30-60 | 30-60 |
| The price of SOOC(¥) | 50 | **70** |
| The increasing ratio of online medical insurance compared to offline | Increase by 10% | **Invariant** |
| The convenience of applying SOOC services | Neutral | Neutral |
| Doctors’ recommendation rate | Weakly  recommend | **Highly**  **recommend** |
|  | □ | □ |

**Choice Set 5：**Which option would you prefer from the following choice? Please tick your choice.

| Attributes | Option A | Option B |
| --- | --- | --- |
| Disease severity | Medium | **Mild** |
| One way trip distance to hospital (min) | 30-60 | **More than 60** |
| The price of SOOC(¥) | 50 | 50 |
| The increasing ratio of online medical insurance compared to offline | Increase by 10% | **Increase by 20%** |
| The convenience of applying SOOC services | Neutral | Neutral |
| Doctors’ recommendation rate | Weakly  recommend | **Recommend** |
|  | □ | □ |

**Choice Set 6：**Which option would you prefer from the following choice? Please tick your choice.

| Attributes | Option A | Option B |
| --- | --- | --- |
| Disease severity | Medium | **Mild** |
| One way trip distance to hospital (min) | 30-60 | **More than 60** |
| The price of SOOC(¥) | 50 | **70** |
| The increasing ratio of online medical insurance compared to offline | Increase by 10% | Increase by 10% |
| The convenience of applying SOOC services | Neutral | **Inconvenient** |
| Doctors’ recommendation rate | Weakly  recommend | Weakly recommend |
|  | □ | □ |

**Choice Set 7：**Which option would you prefer from the following choice? Please tick your choice.

| Attributes | Option A | Option B |
| --- | --- | --- |
| Disease severity | Medium | Medium |
| One way trip distance to hospital (min) | 30-60 | **Within 30** |
| The price of SOOC(¥) | 50 | **30** |
| The increasing ratio of online medical insurance compared to offline | Increase by 10% | **Increase by 20%** |
| The convenience of applying SOOC services | Neutral | Neutral |
| Doctors’ recommendation rate | Weakly  recommend | **Highly recommend** |
|  | □ | □ |

**Choice Set 8：**Which option would you prefer from the following choice? Please tick your choice.

| Attributes | Option A | Option B |
| --- | --- | --- |
| Disease severity | Medium | Medium |
| One way trip distance to hospital (min) | 30-60 | **Within 30** |
| The price of SOOC(¥) | 50 | **70** |
| The increasing ratio of online medical insurance compared to offline | Increase by 10% | **Invariant** |
| The convenience of applying SOOC services | Neutral | **Convenient** |
| Doctors’ recommendation rate | Weakly  recommend | Weakly recommend |
|  | □ | □ |

**Choice Set 9：**Which option would you prefer from the following choice? Please tick your choice.

| Attributes | Option A□ | Option B□ | |
| --- | --- | --- | --- |
| Disease severity | Medium | Medium | |
| One way trip distance to hospital (min) | 30-60 | 30-60 | |
| The price of SOOC(¥) | 50 | **70** | |
| The increasing ratio of online medical insurance compared to offline | Increase by 10% | **Increase by 20%** | |
| The convenience of applying SOOC services | Neutral | **Inconvenient** | |
| Doctors’ recommendation rate | Weakly  recommend | **Recommend** | |
|  | □ | | □ |

**Choice Set 10：**Which option would you prefer from the following choice? Please tick your choice.

| Attributes | Option A | Option B |
| --- | --- | --- |
| Disease severity | Medium | Medium |
| One way trip distance to hospital (min) | 30-60 | **More than 60** |
| The price of SOOC(¥) | 50 | **30** |
| The increasing ratio of online medical insurance compared to offline | Increase by 10% | Increase by 10% |
| The convenience of applying SOOC services | Neutral | **Very**  **convenient** |
| Doctors’ recommendation rate | Weakly  recommend | **Recommend** |
|  | □ | □ |

**Choice Set 11：**Which option would you prefer from the following choice? Please tick your choice.

| Attributes | Option A | Option B |
| --- | --- | --- |
| Disease severity | Medium | Medium |
| One way trip distance to hospital (min) | 30-60 | **More than 60** |
| The price of SOOC(¥) | 50 | 50 |
| The increasing ratio of online medical insurance compared to offline | Increase by 10% | **Invariant** |
| The convenience of applying SOOC services | Neutral | **Inconvenient** |
| Doctors’ recommendation rate | Weakly  recommend | **Highly recommend** |
|  | □ | □ |

**Choice Set 12：**Which option would you prefer from the following choice? Please tick your choice.

| Attributes | Option A | Option B |
| --- | --- | --- |
| Disease severity | Medium | **Severe** |
| One way trip distance to hospital (min) | 30-60 | **Within 30** |
| The price of SOOC(¥) | 50 | 50 |
| The increasing ratio of online medical insurance compared to offline | Increase by 10% | **Increase by 20%** |
| The convenience of applying SOOC services | Neutral | **Inconvenient** |
| Doctors’ recommendation rate | Weakly  recommend | Weakly recommend |
|  | □ | □ |

**Choice Set 13：**Which option would you prefer from the following choice? Please tick your choice.Attributes

|  | Option A | Option B |
| --- | --- | --- |
| Disease severity | Medium | **Severe** |
| One way trip distance to hospital (min) | 30-60 | **Within 30** |
| The price of SOOC(¥) | 50 | **70** |
| The increasing ratio of online medical insurance compared to offline | Increase by 10% | Increase by 10% |
| The convenience of applying SOOC services | Neutral | Neutral |
| Doctors’ recommendation rate | Weakly recommend | **Recommend** |
|  | □ | □ |

**Choice Set 14：**Which option would you prefer from the following choice? Please tick your choice.

| Attributes | Option A | Option B |
| --- | --- | --- |
| Disease severity | Medium | **Severe** |
| One way trip distance to hospital (min) | 30-60 | 30-60 |
| The price of SOOC(¥) | 50 | **30** |
| The increasing ratio of online medical insurance compared to offline | Increase by 10% | Increase by 10% |
| The convenience of applying SOOC services | Neutral | **Inconvenient** |
| Doctors’ recommendation rate | Weakly recommend | **Highly recommend** |
|  | □ | □ |

**Choice Set 15：**Which option would you prefer from the following choice? Please tick your choice.

| Attributes | Option A | Option B |
| --- | --- | --- |
| Disease severity | Medium | **Severe** |
| One way trip distance to hospital (min) | 30-60 | 30-60 |
| The price of SOOC(¥) | 50 | 50 |
| The increasing ratio of online medical insurance compared to offline | Increase by 10% | **Invariant** |
| The convenience of applying SOOC services | Neutral | **Convenient** |
| Doctors’ recommendation rate | Weakly recommend | **Recommend** |
|  | □ | □ |

**Choice Set 16：**Which option would you prefer from the following choice? Please tick your choice.

| Attributes | Option A | Option B |
| --- | --- | --- |
| Disease severity | Medium | **Severe** |
| One way trip distance to hospital (min) | 30-60 | **More than 60** |
| The price of SOOC(¥) | 50 | **30** |
| The increasing ratio of online medical insurance compared to offline | Increase by 10% | **Invariant** |
| The convenience of applying SOOC services | Neutral | Neutral |
| Doctors’ recommendation rate | Weakly recommend | Weakly recommend |
|  | □ | □ |

**Choice Set 17：**Which option would you prefer from the following choice? Please tick your choice.

| Attributes | Option A | Option B |
| --- | --- | --- |
| Disease severity | Medium | **Severe** |
| One way trip distance to hospital (min) | 30-60 | **More than 60** |
| The price of SOOC(¥) | 50 | **70** |
| The increasing ratio of online medical insurance compared to offline | Increase by 10% | **Increase by 20%** |
| The convenience of applying SOOC services | Neutral | **Convenient** |
| Doctors’ recommendation rate | Weakly recommend | **Highly recommend** |
|  | □ | □ |
